# Communication to the Medical Association of Georgia

**Published:** 1876-11

**Authors:** 

**Affiliations:** Oakland, Ga.


THE following correspondence, addressed to the State Medical Association, was received for publication some weeks ago, but too late for the October number of the JOURNAL.

While the caustic irony of the writer may be considered too irritating for a first application, we nevertheless cheerfully give space for discussion, pro and con, on this and other subjects of general interest:

*To the President and members of the Medicat Association of the State of Georgia:*

It appears from your published Transactions for the year 1876, that hereafter every member of said Association must pay annually $5, or be "dropped" out of it. This certainly is just as it ought to be, in regard to all willful delinquents, if, indeed, any man who desires and deserves membership can be willfully delinquent in such a matter. Now, not having had the privilege of attending your last session, nor the honor of aiding in the parturience of said rule, I, by these presents, accord to it my part of its paternity and approval, for the following (among other) reasons:

1st. It puts all members on a dead level; no distinction between rich and poor, denizens of country, town, or city; all pay alike. Moreover, it is coercive, not voluntary, as formerly; a test of obedience, like the apple we read of, that Eve ate. It is beautifully democratic; for no one will know that the poor brother has been just able to squeeze out the five dollars, nor that the rich one don't miss it from his ample stores. Pay up or tumble out from the pale of our enlightenment, is the word, and thus serve as a visible victim of "Locke on Human Understanding," opening only to the talismanic key of five dollars. He that is down and dark, keep him down and dark, *until* the cash comes. He that is up and rising, keep him up and rising, *as long* as the cash comes. No man has any inherent right to be poor, and keep good company—two luxuries that don't grow on the same tree.

2d. No doctor of medicine who is as regularly educated as *we all are*, has any inherent right to be too poor to spare five dollars per annum to so good a cause, nor so foolish as to spend his time, money, and physic on the afflicted poor, of all colors, qualities, and conditions, in remote and dark corners and quarters of our gloriously reconstructed commonwealth.

3d. He has no right to live a day's journey, or more, from any of the great commercial, literary, and scientific centres, and far away from any railroad leading to the seat of our annual literary, medical, gastronomic, and terpsichorean festivals, where so much is said, seen, done, and eaten, equally to interest, instruct, inspire, and fatten us overworked, underpaid, and lean Cassiuses from the country.

4th. We ought not to have allowed to come over us the social and political revolution that has turned things topsy-turvey, and reduced opulence to poverty, and poverty to starvation. Formerly, every man that owned a nigger was able to pay his doctor. Now the nigger won't pay because he is free, and the quondam master can't pay because he has lost his nigger.

5th. He has no right to spend so much of his time and money in doing good to God's poor, in hovels and dark parts of the land, as not to have five dollars left to pay his annual assessment to an institution that is an honor to the medical profession and a blessing to mankind.

6th. He has no right to beget from seven to twelve children *at home* (for we are a fruitful folk), and thus consuming on them all the fruit of his labors, so as to have no extra five dollars to be applied as aforesaid, in elevating an honorable avocation, by printing the products of praiseworthy proceedings in the "Transactions."

7th. But not to bear too heavily on our professional "poor kin"—our brotherhood of the country—let us see what can be said or done for them; for poverty is not always a crime, nor non-payment of debts always dishonesty. Though poor, like most of us, they are an honor to the profession and a blessing to the public. They love their calling, and hate charlatanry in all of its forms. So, please don't cast us out among the herd of uncircumcised root doctors, steam doctors, urine doctors, cancer doctors, homœopaths, nostrum vendors, conjurors, *et id genus omne*, who append a cheap M.D. to their names. Don't put us out in this galaxy, but honor our motives, morals, labors, and orthodoxy with a better reward. Run a line through your congregated body; give us the back seats whenever we *can* attend; let us read the Transactions second hand, when we lack the cash to procure the coveted luxury fresh from the press. But if you prefer (as beggers must not be choosers) keep us *in* as " poor kin " at *half price*, which would be less mortifying to our pride than expulsion for the crime of poverty, or allow us honorably to withdraw from an institution in which we had hoped to live and die.

In conclusion, I may say he is the best friend of the Association who corrects its errors, suggests improvements, and extends its benefits to the greatest number, whether they be rich or poor, far or near, learned or unlearned, distinguished or obscure.

A. Convert, M.D.

*Oakland, Ga.*

