# Errata

**Published:** 1876-12

**Authors:** 


In the correspondence of our November No., addressed to the Medical Association of Georgia, from "A Couvert, M.D.," he was represented as "A. Convert M.D.," through the mistake of taking the *u* for an *n*, and the article *a* for the abbreviation of the first name. He is a *couvert*, or concealed M.D., of Oakland, Ga.

